# Positive affect predicts enhanced quality of life and glycemic outcomes in adolescents with type 1 diabetes

**DOI:** 10.1093/abm/kaag023

**Published:** 2026-05-07

**Authors:** Maya Jayaram, Charity Davis, Kashope Anifowoshe, James C Slaughter, Sarah S Jaser

**Affiliations:** Department of Pediatrics, Vanderbilt University Medical Center, Nashville, TN 37232, United States; Department of Pediatrics, Vanderbilt University Medical Center, Nashville, TN 37232, United States; Department of Pediatrics, Vanderbilt University Medical Center, Nashville, TN 37232, United States; Department of Pediatrics, Vanderbilt University Medical Center, Nashville, TN 37232, United States; Department of Pediatrics, Vanderbilt University Medical Center, Nashville, TN 37232, United States

**Keywords:** positive affect, type 1 diabetes (T1D), adolescents, hemoglobin A1C, health-related quality of life, diabetes distress

## Abstract

**Background:**

Adolescents with type 1 diabetes (T1D) often face challenges with diabetes management and experience diabetes distress related to the burden of treatment. Positive affect, including feelings of enthusiasm, joy, and engagement, may play a role in improving health outcomes.

**Purpose:**

This study aimed to identify demographic and clinical factors associated with positive affect in adolescents with T1D and assess the impact of adolescents’ positive affect on glycemic outcomes and quality of life over time.

**Methods:**

The current study is a longitudinal analysis of data from a multisite randomized controlled trial. Adolescents’ self-reported positive affect was analyzed in relation to demographic variables, clinical outcomes, and diabetes-related quality of life over a 12-month period.

**Results:**

In our sample of 196 adolescents with T1D, greater adolescent age was significantly associated with lower levels of positive affect. Importantly, positive affect was associated with lower HbA1c and higher quality of life over 12 months, after adjusting for baseline levels and demographic factors.

**Conclusions:**

These findings suggest that positive affect may be a protective factor for adolescents with T1D, underscoring the potential for incorporating approaches that induce positive affect as a way to improve diabetes management.

## Introduction

Type 1 diabetes (T1D) is one of the most common childhood chronic health conditions,^1^ and it requires frequent self-management behaviors, including monitoring blood glucose levels, insulin administration, and attention to dietary intake and physical activity.^2^ Adolescents often struggle with these demands due to various barriers, including the physical and psychological changes that occur during this developmental stage.^3^ Additionally, lower rates of consistent diabetes device usage and a lack of support from family and peers further exacerbate these challenges.^4^^,^^5^

Positive affect—feelings of enthusiasm, joy, and engagement—has been identified as an important psychological factor that can improve health outcomes, including those related to chronic conditions such as diabetes.^6–8^ In a longitudinal study of adults with type 2 diabetes, positive affect was found to be a prospective predictor of HbA1c over time.^9^ It is also important to note that negative affect, while related to positive affect, is not its opposite, nor are they mutually exclusive.^10^ Research supports that positive affect, which can be induced,^11^ has potential to promote the use of adaptive coping strategies, enhance adherence to treatment regimens, and improve quality of life.^6^ According to Fredrickson’s “broaden-and-build” theory, positive emotions and affect can allow for broadened attention and the use of more adaptive coping strategies,^12^ which could be beneficial for adolescents as they navigate the challenges of managing T1D.^13^ Despite the known challenges faced by adolescents with T1D, few studies have examined the role of positive affect in this population, and existing studies have been limited in the representativeness of the study samples.^8^^,^^13^ Thus, we sought to identify demographic and clinical factors associated with positive affect in adolescents with T1D, such as sex and age,^14^ and examine the associations between positive affect and key diabetes outcomes, including HbA1c and health-related quality of life, over a 12-month period. We hypothesized that positive affect would be positively associated with lower HbA1c and higher scores on the health-related quality of life scale over time.

## Methods

### Study design and participants

The current study is a secondary analysis of data from a multisite randomized controlled trial aimed at reducing diabetes distress in adolescents with T1D (clinicaltrials.gov identifier: NCT02984709). Participants were recruited from 2 pediatric diabetes centers between 2019 and 2022 in the Southeast and Mid-Atlantic regions. Recruitment was conducted in person at clinic visits until March 2020, after which it was completed remotely. Adolescents were eligible if they were between the ages of 13 and 17, had been diagnosed with T1D for at least 12 months, reported moderate distress (PAID-T score ≥34, or above the median score of adolescents who screened), spoke and read English; and had a device with texting capabilities. Survey data and glycemic data were collected at baseline, 6 months, and 12 months.^15^ Since we did not find intervention effects on quality of life or HbA1c,^15^ we included all randomized participants in this secondary analysis (*n* = 196). Details of the participant demographics are available in Table 1.

**Table 1:** Demographic and clinical characteristics of participants (*n* = 196).

Adolescent age in years (mean [SD])	15.3 [1.4]
Child sex, female (*N* [%])	113 [58]
Child race and ethnicity, (*N* [%])	
Non-Hispanic White	122 [62]
Non-Hispanic Black	46 [23]
Other race or ethnicity	28 [14]
Annual household income category (*N* [%])	
<$24 999	16 [8]
$25 000-49 999	35 [18]
$50,000-89,999	47 [24]
$90 000-149 999	42 [21]
≥$150 000	56 [29]
Device use (*N* [%])	
Continuous glucose monitor	160 [82]
Insulin pump	115 [59]
Diabetes duration in years (mean [SD])	6.3 [3.7]
HbA1c (mean [SD])	9.1% [2.2]

Some participants used both a continuous glucose monitor and an insulin pump.

### Measures

Adolescents’ affect was assessed using the Positive and Negative Affect Schedule for Children (PANAS-C), which measures the frequency of positive and negative emotions experienced by participants.^16^ The Positive Affect scale consists of 12 items [α = .89], and the Negative Affect scale consists of 15 items [α = .88]. Summed scores were calculated, and higher scores indicate higher levels of affect.

Health-related quality of life was evaluated using the Type 1 Diabetes and Life (T1DAL) questionnaire, a tool that captures the impact of diabetes on daily living and psychosocial well-being.^17^ While some items ask about worry, stress, or frustration related to diabetes management, no items directly overlap with the terms used in the PANAS. Scaled scores range from 0 to 100, and higher scores indicate better quality of life [α = .79].

Hemoglobin A1C point-of care values were obtained from adolescents’ medical records when available (72.3% of participants). Given that the study occurred during the start of the COVID-19 pandemic, some participants were unable to attend in-person diabetes clinic visits. In those cases, we sent them an at-home HbA1c kit (22.3%). If they did not return the kit, we used data from their continuous glucose monitor for an estimated value (Glucose Management Indicator, 5.3%). These alternative measures are highly correlated with point-of-care values and were recommended for telemedicine visits.^18^

Additional demographic and clinical variables, including adolescent age, sex, race/ethnicity, household income, diabetes device use (continuous glucose monitor, insulin pump), and the duration of the T1D diagnosis, were collected through caregiver questionnaires.

### Data analysis

We analyzed all randomized participants using listwise deletion at the model level, without multiple imputation. Participants contributed data at all available time points (baseline, 6 months, 12 months), and analyses were conducted under the assumption that data are missing at random. Descriptive statistics were used to summarize demographic and clinical characteristics. Independent-sample *t*-tests examined sex differences in positive affect. Spearman correlation coefficients assessed the relationships between positive and negative affect, age, diabetes duration, HbA1c values, and health-related quality of life, as these variables have previously been found to be associated with positive affect.^13^^,^^14^

The association between baseline PANAS positive affect and each longitudinal outcome (A1C, T1DAL) was estimated using linear mixed effects models fitted via restricted maximum likelihood (REML). Time was treated as a categorical factor (baseline, 6 months, 12 months). Unadjusted models included only PANAS positive affect, time, and their interaction. Adjusted models additionally controlled for baseline PANAS negative affect, age at enrollment, and sex (female vs. male). The PANAS-by-time interaction allows the slope of the outcome on baseline PANAS positive affect to differ across time points. All inference was based on CR2 bias-reduced linearization cluster-robust sandwich variance estimators with Satterthwaite small-sample degrees of freedom. A joint Wald test of all PANAS positive-related coefficients (main effect and interactions) was used to assess the overall association. Time-specific slopes were obtained as linear combinations of the model coefficients with corresponding CR2-based confidence intervals. Results are summarized as time-specific slopes that are linear combinations of the model coefficients of the main effect at baseline plus relevant interactions at 6 and 12 months per 10-point increase in PANAS score.

## Results

The current study included 196 adolescent participants. The demographic and clinical characteristics of the sample are included in Table 1.

### Demographic and clinical factors associated with positive and negative affect

There was no significant difference between males and females in baseline levels of positive affect (*M*_male_ = 36.6, *M*_female_ = 34.1, *t *= 1.96, *P* = .052, Cohen’s *d* = .28) or negative affect (*M*_male_ = 32.4, *M*_female_ = 34.4, *t *= −1.28, *P* = .201, Cohen’s *d* = −.19). Adolescent age was associated with lower positive affect scores at baseline (*r *= −0.25, *P* <.001) but not with negative affect (*r* = .06). Diabetes duration was not significantly associated with positive or negative affect. No significant associations were found between adolescents’ affect and continuous glucose monitor usage, insulin administration type, race/ethnicity, or income.

As seen in Table 2, adolescents’ positive affect scores were associated with significantly lower HbA1c values at 12 months (*r *= −0.18, *P* = .019), but not with HbA1c values at baseline or 6 months. Positive affect scores were significantly associated with higher health-related quality of life at baseline (*r* = 0.29), 6 months (*r* = .0.24), and 12 months (*r* = 0.24). Negative affect was significantly associated with lower health-related quality of life across all 3 time points (*r *= −0.50, −0.27, and −0.23, all *P* < .001), but not with HbA1c.

**Table 2:** Spearman’s correlations between positive affect, demographic characteristics, and diabetes outcomes over 12 months.

	1.	2.	3.	4.	5.	6.	7.	8.	9.	10.
Positive affect	—									
Negative affect	−.25^a^	—								
Diabetes duration	−.08	.01	—							
Adolescent age	−.23^a^	.06	.15	—						
HbA1c baseline	−.13	.08	.18^b^	−.04	—					
HbA1c 6 months	−.17	.09	.15	−.01	.74^a^	—				
HbA1c 12 months	−.19^b^	.03	.10	.01	.72^a^	.75^a^	—			
T1DAL baseline	.29^a^	−.50^a^	−.06	−.11	−.12	−.11	−.17^b^	—		
T1DAL 6 months	.24^a^	−.27^a^	−.05	.04	−.06	−.08	−.16	.47^a^	—	
T1DAL 12 months	.24^a^	−.23^a^	.01	.08	−.08	−.17^b^	−.24^c^	.48^a^	.70^a^	—

Abbreviation: T1DAL, type 1 diabetes and life.

a *P* < .001.

b *P *< .05.

c *P* < .01.

### Positive affect as a predictor of glycemic outcomes and quality of life

As seen in Table 3, adolescents’ self-reported baseline level of positive affect was significantly associated with HbA1c at 12 months in the unadjusted and adjusted model. Controlling for negative affect, adolescent age and sex, adolescents with a 10 point higher positive PANAS at baseline were expected to have a 0.48% lower HbA1c at 12 months (*β* = −.48, *P* = .007). In addition, baseline positive affect was significantly associated with diabetes-related quality of life at baseline and 12 months in the unadjusted model. Controlling for negative affect, adolescent age and sex, adolescents with a 10 point higher positive PANAS at baseline were expected to have a 2.57 higher T1DAL score 12 months later (*β* = 2.57, *P* = .026).

**Table 3:** Associations between baseline positive affect and diabetes outcomes—unadjusted and adjusted linear mixed effects models.

	HbA1c unadjusted		HbA1c adjusted	
Variable	Estimate (95% CI)	*P* value	Estimate (95% CI)	*P* value
PANAS positive affect				
Baseline	−0.29 (−0.67 to 0.09)	.131	−0.29 (0.67 to 0.09)	.134
6 Months	−0.36 (−0.76 to 0.03)	.070	−0.36 (−0.75 to 0.03)	.071
12 Months	−0.48 (−0.82 to −0.14)	.006	−0.48 (−0.82 to 0 − 0.14)	.007
PANAS negative affect		—	0.10 (−0.19 to 0.38)	.503
Age (years)		—	−0.06 (−0.26 to 0.14)	.544
Female		—	−0.06 (−0.62 to 0.51)	.842
	T1DAL unadjusted		T1DAL adjusted	
Variable	Estimate (95% CI)	*P* value	Estimate (95% CI)	*P* value
PANAS positive affect				
Baseline	4.11 (2.03 to 6.19)	<.001	2.85 (0.96 to 4.74)	.004
6 Months	3.51 (1.00 to 6.01)	.007	2.21 (−0.15 to 4.57)	.066
12 Months	3.86 (1.49 to 6.22)	.002	2.57 (0.32 to 4.83)	.026
PANAS negative affect		—	−.3.71 (−5.19 to −2.23)	<.001
Age (years)		—	0.83 (−0.25 to 1.91)	.132
Female		—	−5.01 (−7.98 to −2.03)	.001

The time-specific slopes represent the expected change in Hba1c (%-points) or T1DAL score associated with a 10-point increase in baseline PANAS positive score at each assessment period.

Abbreviations: PANAS, positive and negative affect scale; T1DAL, type 1 diabetes and life.

## Discussion

The results of this longitudinal study provide evidence for the protective effects of positive affect in adolescents with T1D. The consistent associations between higher positive affect with lower HbA1c and higher health-related quality of life over time underscore the potential for incorporating approaches that induce positive affect in adolescent diabetes management. By controlling for negative affect in this analysis, similar to Horner et al’s study in adults with diabetes,^9^ we were able to rule out other potentially confounding factors. Given that negative and positive affect are not mutually exclusive and can therefore co-occur, adjusting for its presence amongst participants in our sample allowed us to focus on the impact of positive affect.

In our sample, the negative correlation between adolescent age and positive affect suggests that older adolescents with T1D may face additional challenges in maintaining a positive outlook. Previous research highlights changes in emotional regulation and expression during adolescence as potential contributors to these challenges.^19^ This finding emphasizes the need for ongoing support and interventions that may promote positive affect throughout adolescence and as individuals navigate the long-term management of their condition. It is also interesting to note that there were no sex differences in positive or negative affect, which could be due to the adolescents in the study being selected based on their elevated levels of diabetes distress. Future studies could explore interactions between age and sex on positive affect.

Our findings that adolescents’ positive affect was significantly associated with HbA1c and quality of life 12 months later provides support for the potential benefits of fostering positive emotions in adolescents with T1D. Interventions designed to boost positive affect could have far-reaching impacts on both physical health outcomes and overall well-being.^6^ As such, healthcare providers may consider incorporating assessments of positive affect and recommending evidence-based strategies to boost positive affect, such as gratitude practices, to enhance routine care for adolescents with T1D.^20^^,^^21^ This attention to positive affect could potentially lead to improved long-term health outcomes and psychosocial well-being.

Limitations of the current study must be acknowledged. First, the participants in this study were adolescents who agreed to enroll in a 12-month clinical trial. Given this level of commitment, the current sample may not be representative of the general population of adolescents with T1D. Secondly, our sample includes a limited age range (ages 13-17) and the adolescents were selected based on experiencing diabetes distress, so these findings regarding positive affect may not generalize to other youth with T1D. Thirdly, the associations between positive affect and quality of life may be inflated by shared method variance, as adolescents were self-reporting on both. Finally, although we report that positive affect was associated with glycemic outcomes and quality of life 12 months later, it may be the case that other factors (ie, demographic factors or differences in diabetes management) influenced these outcomes over time. The study took place during COVID-19 pandemic, which also may have affected outcomes.

The current study demonstrates that positive affect is important to consider and may serve as a protective factor for adolescents with T1D. If, as hypothesized, positive affect is related to the use of more adaptive coping strategies and more engagement in diabetes management,^6^^,^^12^ there may be a delayed effect of positive affect on diabetes-related outcomes, particularly HbA1c, since it is a measure of average blood glucose levels over the previous 8-12 weeks. Incorporating positive psychology approaches in adolescent diabetes management could significantly enhance health outcomes and psychosocial well-being over the long term. Future research should focus on developing and implementing tailored strategies to boost positive affect in routine adolescent care for T1D, potentially leading to improved disease management and health-related quality of life for this vulnerable population.

## Data Availability

De-identified data from this study are not available in a public archive. De-identified data will be made available (as allowable according to institutional IRB standards) by emailing the corresponding author.

## Analytic code availability

There is no analytic code associated with this study.

## Materials availability

Some of the materials used to conduct the study are presented in a public archive: NCT03845465.

## Study registration

The study was pre-registered at ClinicalTrials.gov and clinical trial identification number: NCT03845465.

## Analytic plan pre-registration

The analysis plan for this secondary analysis was not formally pre-registered.
